# Transposable elements: genome innovation, chromosome diversity, and centromere conflict

**DOI:** 10.1007/s10577-017-9569-5

**Published:** 2018-01-13

**Authors:** Savannah J. Klein, Rachel J. O’Neill

**Affiliations:** Institute for Systems Genomics and Department of Molecular and Cell Biology, University of Connecticut, Storrs, CT 06269 USA

**Keywords:** Centromeric retroelement, Satellite, Transposable element, TE, Genome defense, Chromosome evolution, Conflict

## Abstract

Although it was nearly 70 years ago when transposable elements (TEs) were first discovered “jumping” from one genomic location to another, TEs are now recognized as contributors to genomic innovations as well as genome instability across a wide variety of species. In this review, we illustrate the ways in which active TEs, specifically retroelements, can create novel chromosome rearrangements and impact gene expression, leading to disease in some cases and species-specific diversity in others. We explore the ways in which eukaryotic genomes have evolved defense mechanisms to temper TE activity and the ways in which TEs continue to influence genome structure despite being rendered transpositionally inactive. Finally, we focus on the role of TEs in the establishment, maintenance, and stabilization of critical, yet rapidly evolving, chromosome features: eukaryotic centromeres. Across centromeres, specific types of TEs participate in genomic conflict, a balancing act wherein they are actively inserting into centromeric domains yet are harnessed for the recruitment of centromeric histones and potentially new centromere formation.

## Introduction

Transposable elements (TE) are segments of DNA that can move, or transpose, within the genome. The existence of elements capable of intragenomic mobility was first discovered in maize by American scientist Barbara McClintock in the 1940s and described in her seminal 1950 paper (McClintock 1950). Originally dismissed as an obscure observation, McClintock’s work was eventually recognized as groundbreaking, challenging the view of the genome as a static unit of heritability, and leading to the emergence of the concept of the “dynamic genome.” Following McClintock’s discovery, TEs were viewed merely as “junk DNA” and “selfish DNA parasites,” simple sequences that multiply within the genome yet provide no apparent beneficial contribution to its host (Doolittle and Sapienza 1980; Orgel and Crick 1980). However, genome-scale studies over the past several decades have shown that TEs play a key role in genome function, chromosome evolution, speciation, and diversity.

The Human Genome Project revealed just how abundant TEs are in humans, making up approximately 45% of the overall human genome content (Cordaux and Batzer 2009; Lander et al. 2001). TEs can be divided into two major classes based on transposition mechanism: DNA transposons, which move via a “*cut*-and-paste” mechanism and RNA transposons, also referred to as retrotransposons or retroelements, which move via a “*copy*-and-paste” mechanism. Retroelements can then be further subdivided into long terminal repeat elements (LTRs), including retroviruses, and non-LTR elements. While there is no evidence for DNA transposon activity in humans in the past 50 million years (Lander et al. 2001), some retroelements are still active today, including members of the non-LTR class of retroelements, namely long interspersed nuclear elements (LINEs), short interspersed nuclear elements (SINEs), SINE-VNTR-*Alu* elements (SVAs) (Mills et al. 2007), and potentially members of the LTR-class of endogenous retroviruses (HERVs). LINEs are considered the only autonomous non-LTR TE in humans since these TEs encode all of the components required for transposition, while SINEs and SVAs are considered non-autonomous as these elements require the presence of another active TE to mobilize (Dewannieux et al. 2003). Within the LINE and SINE retroelement classes in humans, two distinct families stand out: LINE1 and Alu, respectively. LINE1s, the only remaining mobile LINE family in humans, constitutes ~ 17–20% of the human genome (Lander et al. 2001). Alus, the active and mobile SINE family in humans, constitutes a smaller portion of the human genome (~ 11%) by nucleotide count, yet are more abundant in copy number than LINE1s due to their 20-fold smaller element size (Cordaux and Batzer 2009; Quentin 1992; Roy-Engel et al. 2002). In contrast to LINE1 and Alu, SVAs only make up ~ 0.2% of the human genome (Cordaux and Batzer 2009; Wang et al. 2005).

A caveat to the observation that mobile TEs in humans are restricted to LINE1s, Alus, and SVAs was recently discovered when members of the human endogenous retrovirus family HERV-Ks, also known as HML2s (~ 1% of the human genome (Subramanian et al. 2011)), were found to contain full, intact open reading frames and were identified in polymorphic sites in the human population, implicating recent, if not retained, mobility (Belshaw et al. 2005; Belshaw et al. 2004; Dewannieux et al. 2006; Hughes and Coffin 2004). With rare exceptions, TEs are found in the genomes of nearly all eukaryotic species. However, the TE composition within the genome and the types of active elements are highly variable among species (see Huang et al. 2012 and Sotero-Caio et al. 2017 for reviews). This review focuses on the impact of TEs on chromosome function and evolution, with an emphasis on the human genome and the retroelements that retain the capacity to mobilize. Furthermore, this review examines the contribution TEs have on a discrete functional domain in the eukaryote genome, the centromere.

## Structure and transposition of active TEs in the human genome

A full-length LINE1 (~ 6 kb) consists of a 5′ UTR with a bidirectional RNA polymerase II promoter, two open reading frames (ORF-1 and ORF-2), a 3′ UTR, and a polyadenylation signal followed by a poly-A tail (Fanning and Singer 1987a; Fanning and Singer 1987b). The bidirectional promoter not only allows for the expression of the LINE1 and its two internal ORFs but also promotes antisense transcription of the 5′ UTR and, at least in primates, an open reading frame (ORF-0) that carries the potential to create fusion genes with upstream regions in the genome (Denli et al. 2015). ORF-1 codes for a protein with RNA-binding capabilities and nucleic acid chaperone activity, while ORF-2 codes for a protein with endonuclease and reverse transcriptase (RT) activity (Dai et al. 2014).

A full-length Alu (~ 300 bp) is derived from the signal recognition particle RNA 7SL (Ullu and Tschudi 1984) and consists of two similar monomers with an A-rich linker in-between, A- and B-boxes present in the 5′ monomer, and a poly-A tail lacking the preceding polyadenylation signal resulting in an elongated tail (up to 100 bp in length) (Quentin 1992; Roy-Engel et al. 2002). Alus can be transcribed by RNA polymerase III using the internal promoters within the A- and B-boxes; however, Alus contain no ORFs and therefore do not encode for protein products (Panning and Smiley 1993; Sawada et al. 1985).

A full-length SVA (SINE-VNTR-*Alu*) element (~ 2–3 kb) is a composite unit (Wang et al. 2005) that contains a CCCTCT repeat, two *Alu*-like sequences, a VNTR, a SINE-R region with *env* (envelope) gene, the 3′ LTR of HERV-K10, and a polyadenylation signal followed by a poly-A tail (Ostertag et al. 2003; Wang et al. 2005). It is most likely that SVAs are transcribed by RNA polymerase II, although it is unknown whether SVA elements carry an internal promoter (Wang et al. 2005).

A full-length HERV-K element (~ 9–10 kb) is comprised of ancient remnants of endogenous retroviral sequences (Ono 1990) and includes two flanking LTR regions surrounding three retroviral ORFs: (1) *gag* encoding the structural proteins of a retroviral capsid; (2) *pol-pro* encoding the enzymes: protease, RT, and integrase; and (3) *env* encoding proteins allowing for horizontal transfer (Alazami et al. 2004; Dewannieux et al. 2005). The LTR of HERV-K contains an internal, bidirectional promoter that appears to be under the transcriptional control of RNA polymerase II (Domansky et al. 2000; Leupin et al. 2005).

Despite the observation that some mobile elements are still capable of encoding for proteins that facilitate mobility, it is the RNA transcript of a retroelement that is an integral component of its transposition via reverse transcription. For example, LINE1 is transcribed in the nucleus, after which both nascent LINE1 RNA and its translated protein form a ribonucleoprotein protein complex (RNP) in the cytoplasm. The RNP complex migrates back into the nucleus, where the ORF2 protein, containing endonuclease (EN) activity, makes a nick in genomic DNA at an insertion site. ORF2 also encodes for RT, which converts the RNA to DNA via target primed reverse transcription (TPRT). The result of this RT-mediated movement is the insertion of a full-length, or often 5′ truncated, LINE1 into the genome in a novel location (Morrish et al. 2002).

The retrotransposition of Alu also requires an RNA-intermediate, but the lack of ORFs renders it reliant on the RT and EN proteins encoded by an autonomous TE (e.g., LINE1) (Dewannieux et al. 2003). SVA mobility is also driven *in trans* by LINE1 machinery (Raiz et al. 2012). Unlike SINEs, SVAs, and LINEs, the activity of HERV-K elements is guided by proteins encoded within the HERV genome; namely, *gag*, *pol*, *pro*, and *env* (Boller et al. 1993; Lower et al. 1993; Lower et al. 1995). Integration of members of all four active TE families results in target site duplications (TSDs), duplications of a short sequence segment of genomic DNA upon insertion, which vary in size based on the element (Craig 1995).

## Genome defense mechanisms (genome vs TEs)

While the four known TE families that contain active elements within the human genome collectively comprise almost 30% of the total genome content, only a very small portion of TEs within these families, less than 0.05%, of elements retain the ability to mobilize (Mills et al. 2007). Active TEs can lose their mobility through stochastic processes, such as the accumulation of mutations that eliminate ORFs or render translated proteins inactive, including single nucleotide changes, insertions, and deletions. TEs also become immobile as the result of their own transposition. For example, the majority of LINEs have been immobilized as the result of 5′ truncation following premature RT termination during the production of dsDNA prior to integration (Alisch et al. 2006). To outpace extinction through mutational inactivation, TE replication must exceed that of the host genome. Thus, TEs are considered “selfish elements” (Doolittle and Sapienza 1980; Orgel and Crick 1980) since they continuously replicate and create new copies of themselves within a host genome as part of their lifecycle, despite the fact that unregulated TE replication can create deleterious effects on a genome, such as insertional mutations and chromosome breakage. Considered by many a classic example of host-invader conflict, TEs that increase in copy number in the germline would spread through a population quickly but mechanisms within host genomes that diminish or eliminate this activity would provide a selective advantage to the host. One would expect a finite lifespan for TEs as selection would appear to favor complete silencing or loss. However, TEs are transmitted through the germline and represent a heritable portion of genomes, rather than existing as a single lifecycle, infectious invader in the classical sense. Thus, TEs and host genome interactions should be considered in the context of the Red Queen’s Hypothesis (Van Valen 1973), wherein TEs and host genomes experience antagonistic coevolution (McLaughlin and Malik 2017). Because of the host-TE conflict, the impact of TEs to genomes extends beyond insertional mutations and includes the evolution of genome defense mechanisms to combat the unfettered TE replication and mobility, as well as examples where TEs provide a selective advantage or are “domesticated.”

As part of this antagonistic coevolution, several different genome defense mechanisms have evolved across eukaryotes to combat TE mobility, targeting TEs at either the *transcriptional level* or *the post-transcriptional level.* Silencing TEs at the transcriptional level involves epigenetic DNA and/or chromatin modifications that can alter the protein accessibility to DNA required for transcription, therefore regulating the transcriptional activity of TEs. While epigenetic modifications are heritable, the TE sequence itself has not been altered in any way and thus, it may retain its ability to mobilize through transcription in the event epigenetic modifications change and the element is reactivated. A multitude of modifications to chromatin exist that would result in the repression of TE transcription. These include the following: modifications to histone tails, methylation of DNA, and alterations of chromatin packaging and condensation (Slotkin and Martienssen 2007). It has been shown that mutations in genes that are required for repressive histone tail modifications lead to TE reactivation; for example, in mice a mutated SUV39 (H3K9 methyltransferase gene) leads to a twofold increase in the number of TE transcripts (Martens et al. 2005). In addition to chromatin modifications, DNA methylation suppresses TE activity in normal cells (Hackett et al. 2012; Ikeda and Nishimura 2015; Reik 2007; Yoder et al. 1997). In fact, there is evidence that the length of CpG islands associated with gene transcription is correlated with the density of LINEs and Alus in the human genome, with a set of “transitional CpGs” acting as a buffer between the hypermethylated, and thus silenced, TE and active gene transcription (Kang et al. 2006). Even the lesser known small RNA class, PIWI-interacting RNAs (piRNAs), has been shown to be essential in the establishment of methylation in the germline to suppress TE activity in offspring (Aravin et al. 2004; Kalmykova et al. 2005; Siomi et al. 2011; Vagin et al. 2004). Furthermore, studies in mammalian embryonic stem (ES) cells have shown that KRAB-zinc finger proteins (KZFP) and their corepressor, TRIM28, are able to induce epigenetic silencing to repress TEs, and hence, regulate their local transcriptional impact in the genome (Jacobs et al. 2014; Rowe et al. 2010; Wolf et al. 2015). Interestingly, the KZFP gene family in primates has been rapidly expanding and evolving to repress TEs when they undergo mutations and mobilize (Jacobs et al. 2014). Lastly, chromatin remodeling proteins have been shown to participate in TE silencing. For example, in the model plant *Arabidopsis thaliana*, the chromatin-remodeling protein DDM1 is essential for the silencing of TEs and the condensation of chromatin (Lippman et al. 2004).

In contrast to targeting transcriptional activity, post-transcriptional regulation of TEs targets the RNA molecules to prevent the RNA transcript from re-integrating into the genome. The main source of this form of regulation is through the RNA interference (RNAi) mechanism. TE transcription can result in the formation of double-stranded RNAs (dsRNAs), which have been shown to trigger RNAi in a wide variety of organisms (Horman et al. 2006). These dsRNAs can be cleaved into small-interfering RNAs (siRNAs), which associate with the RNA-induced silencing complex (RISC) for the targeting of the TE transcripts resulting in transcript cleavage or degradation. *Caenorhabditis elegans* (*C. elegans*) is a prime example of the use of RNAi as a primary mechanism for silencing. In *C. elegans*, Tc1 elements (a type of transposon) give rise to dsRNAs, which are cleaved into siRNAs that can mediate post-transcriptional degradation of the target TE transcript (Ketting et al. 1999; Rosenzweig et al. 1983). In addition, siRNAs have been shown to interact with piRNAs, providing an explanation for observed Tc1 activity in *C. elegans* somatic cells, but not in the germline (Bagijn et al. 2012; Emmons et al. 1983; Phillips et al. 2015; Sijen and Plasterk 2003).

## Impacts of TEs on the genome (TEs vs genome)

**Fig. 1:**
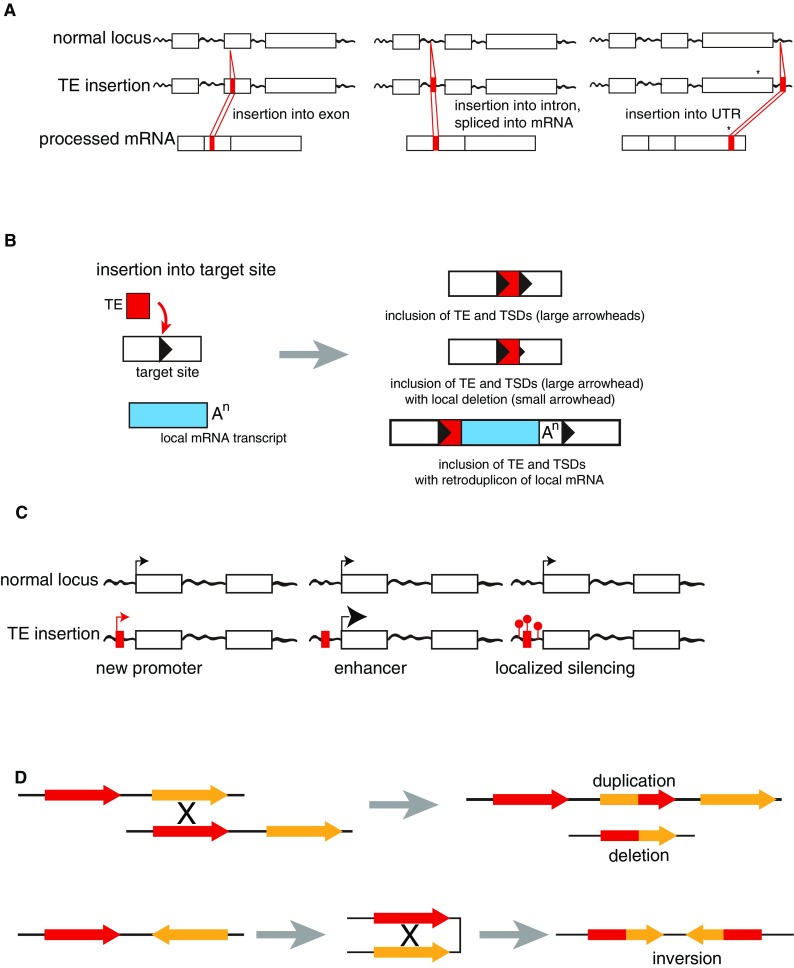
The impact of TEs on the genome. **a** From left: insertion of a TE (red) into an exon and incorporation into the final mRNA; insertion of a TE (red) into an intron and contribution of splice donor and acceptor sites that lead to splicing of the TE into the mRNA; insertion of a TE (red) into a 3′ UTR with concomitant use of an alternative splice donor (asterisk) within the last exon and use of a splice acceptor within the TE, resulting in an alternative 3′ UTR including the TE. **b** Insertion of a TE (red) into a target site (arrowhead) results in various insertional mutations, right. From top: insertion of TE and TSDs; insertion of TE and TSDs with a small deletion in the right TSD; insertion of the TE, TSDs, and a local mRNA transcript (blue) as a retroduplication. **c** Insertion of a TE upstream of a coding region can result in, from left: establishment of a new promoter; enhanced transcription; localized silencing due to methylation of the TE (red lollipops). **d** (Top) NAHR events between two related TEs (red and orange) in tandem on either the same strand or different strands of DNA can result in duplications or deletions. (Bottom) NAHR events between inverted TEs results in an inversion

TEs affect genomes in two major ways: via the mobilization event or post-insertion. The impacts of mobilization are simpler and local; the extent of which is dependent upon the location of the TE insertion site within the genome (Fig. 1). A primary example is seen with insertional mutagenesis, in which insertion of a mobile element results in disruption of a gene. Classic examples of such insertional mutations are the insertions of LINE1 into exon 14 of the factor VIII gene. Each of these insertions resulted in TSDs of portions of the gene, rendering the gene non-functional and triggering hemophilia in patients (Kazazian et al. 1988) (Fig. 1A). As of 2016, there are 124 documented LINE1-mediated insertions that have resulted in genetic disease (Hancks and Kazazian 2016), with LINE1-mediated retrotransposition events accounting for approximately one in every 250 pathogenic human mutations (Wimmer et al. 2011). Insertional mutagenesis can also lead to splice site changes with concomitant alteration to protein structure and/or function, as exemplified by an SVA insertion into the *fukutin* gene which results in abnormal fukutin splicing and the development of Fukuyama muscular dystrophy (FCMD) (Taniguchi-Ikeda et al. 2011) (Fig. 1A). LINE1s have localized impact through the requisite use of target-primed reverse transcription (TPRT), which results in TSDs (Fig. 1B). On occasion, TPRT leads to small deletions of target site DNA and/or the addition of filler DNA at the target site (Lavie et al. 2004; Narita et al. 1993) (Fig. 1B). LINE1 TPRT-induced target site deletions can be as small as a few base pairs, or as large as a megabase in size (Vogt et al. 2014). LINE1 reverse transcription activity can also lead to the insertion of processed mRNAs along with the LINE1, resulting in gene retroduplications (Fig. 1B). While typically non-functional due to a missing nascent promoter, gene retroduplications do lead to genetic diversity and have, in some cases, led to intragenic insertional events that may be linked to disease (Zhang et al. 2017).

The post-insertion impacts of TEs on a genome are more global and as such can significantly influence genome structure, regional function, and chromosome dynamics. For example, TEs act as binding sites for proteins that form the axial elements of the synaptonemal complex, as was demonstrated for actively retrotransposing SINEs in mice and in macaques (Johnson et al. 2013). Moreover, TEs often continue to impact the genomic landscape long after they are transcriptionally inactivated, with variation in insertion sites and timing resulting in functional polymorphism for gene expression (Marcon et al. 2015; Sanseverino et al. 2015). The epigenetic landscape can also be altered by TE insertions, thus affecting the expression of genes surrounding the insertion. TEs tend to be methylated (repressed); therefore, insertion of a mobile element can result in an increase of local levels of DNA methylation or even inactivation of histone tail modifications (Byun et al. 2012). TEs inserted into non-coding regions of genes (introns, upstream, and downstream) can act as alternative promoters, enhancers, or polyadenylation signals for these genes (Fig. 1C). For example, LINE1s have been found in the non-coding regions of ~ 80% of human genes and the density of LINE1s in host genes is inversely correlated with expression of those genes (for reviews see: Chuong et al. 2017; Cohen et al. 2009; Goodier and Kazazian 2008).

Post-insertion impacts also include deletions, segmental duplications, and inversions, all resulting from non-allelic homologous recombination (NAHR), the mispairing of two stretches of highly similar DNA sequences, such as similar TEs (Bailey et al. 2003; Cordaux and Batzer 2009; Deininger and Batzer 1999; Hancks and Kazazian 2012; Lee et al. 2008) (Fig. 1D). An accumulation of these genomic alteration events can lead to various forms of genomic instability, which are associated with many human genetic disorders (for reviews see: Burns 2017; Colnaghi et al. 2011), as well as evolutionary novelty (Brown and O’Neill 2010). Surprisingly, despite being found at very low frequency, there is evidence of TE evolution and novelty within the human population, with Alus providing the highest levels of TE genetic diversity (Rishishwar et al. 2015; Wang et al. 2017a). Wang et al. (2017b) demonstrated that gene expression differences among human individuals result from polymorphisms of Alu, LINE1, and SVA insertion sites after constructing poly-TE genotypes of 10,106 poly-TE insertions and genome-wide expression profiles for 445 individuals. Given that these polymorphic TE insertions “with functional consequences,” in terms of gene expression profiles, are found within a healthy population, TE insertions are not strictly deleterious but may also result in regulatory changes and gene expression variants that may be selected for during human genome evolution (Wang et al. 2017b).

NAHR followed by unequal recombination is most common between Alus, although it has been reported with LINE1 (Han et al. 2008; Sen et al. 2006). Interchromosomal TE recombination may lead to deletions and duplications of the involved chromosomes (Emanuel and Shaikh 2001 and reviewed in Kazazian and Moran 2017), while intrachromosomal recombination can cause deletions, duplications, and inversions (Gilbert et al. 2005; Symer et al. 2002; and reviewed in Beck et al. 2011). Interestingly, a common feature of human Alus is their frequent appearance as inverted repeats (IRs) within the genome. IRs have been shown to form hairpin structures that are prone to double-strand breaks (DSBs) and serve as sites of replication stalling in yeast, bacteria, and mammalian cells (Lobachev et al. 2000; Voineagu et al. 2008) that may also increase local incidents of DNA breaks (Brown et al. 2012). In response to TE-mediated recombination events, several mechanisms have evolved to repair resulting chromosomal structures and prevent further genomic instability. These repair mechanisms involve DNA recombination processes such as single-strand annealing, synthesis-dependent strand annealing, and non-homologous end joining resulting in the formation of these abnormal chromosomal structures (reviewed in Beck et al. 2011).

In some cases, structural changes as a result of TE activity, particularly inversions, can pose reproductive barriers among individuals within interbreeding populations (Brown and O’Neill 2010). For example, comparisons of archaic and modern human genomes indicate a burst of TE activity occurred in the lineage that led to Denisovans, concomitant with an increase in divergent structural rearrangements (Rogers 2015). In addition, genomic loci defined by structural variation were also defined by low rates of introgression from the Neanderthal lineage into the modern human genome, indicating that such rearrangements acted as barriers to gene flow (Rogers 2015).

## The centromere: a high TE impact arena

Structural rearrangements fostered by TEs can affect karyotypic evolution through the derivation of novel chromosome forms and reproductive barriers to gene flow. A functionally defined region of the eukaryotic chromosome shows strong evidence for recurring evolutionary novelty facilitated by TE activity: the centromere. The impact of TEs on centromeres spans both the proteins involved in centromere function and identity as well as the structure of the genomic landscape of the centromere itself.

One of the earlier examples of the relationship between TEs and centromere function is the derivation of the centromere protein CENP-B from the *tcl*/*mariner*/*pogo* family of DNA transposases (Kipling and Warburton 1997). Sharing remarkable protein sequence identity to *Tigger* elements in the *pogo* family (Smit and Riggs 1996), CENP-B binds to a DNA box, termed the CENP-B box, which shows similarities to the terminal inverted repeats (TIRs) that are targeted by *Tigger* for endonucleolytic cleavage and strand transfer to a target location during transposition (Smit and Riggs 1996). CENP-B boxes are found in satellites resident at centromeres in a broad range of species, including humans, mice, giant pandas, and marsupials, prompting the theory that CENP-B promotes nicks in satellites and further facilitates homologous recombination among arrays (Kipling and Warburton 1997). However, to fully appreciate the influence of TEs on centromere formation, maintenance, and diversity, we should consider the factors that define centromere identity and function.

In the strictest sense, the centromere is the chromosomal site of kinetochore formation and spindle attachment. As such, a properly functioning centromere is required for the stable inheritance of each chromosome during mitosis and meiosis, with a disruption of centromere function leading to chromosome loss, breakage, or structural change. Although the requisite role for the centromere in the propagation of genetic material is well conserved across eukaryotes, as are many of the proteins involved in centromere function and kinetochore assembly, rapid evolution among species has been observed for nascent centromeric DNA sequences, overall centromere size, and the centromere proteins that are in direct contact with centromeric DNA (Bulazel et al. 2007; Henikoff et al. 2001; Henikoff and Malik 2002; Malik and Henikoff 2002, 2009; Melters et al. 2013; Zedek and Bures 2012).

Most multicellular eukaryotic centromeres harbor characteristic repeat structures of species-specific satellites (e.g., α satellites in human and minor satellites (miSAT) in mouse). While satellites appear virtually ubiquitous in regional centromeres that are fixed within species (Alkan et al. 2011), several studies support the observation that centromeric satellites are not *sufficient* to form kinetochores (Nakano et al. 2003; Warburton et al. 1997). Thus, the presence of species-specific satellite DNA alone is not the primary determinant for recruiting centromeric histones to a specific chromosomal location. In fact, detailed mapping from ectopic centromeres in humans (e.g., neocentromeres, see below) suggests that satellite DNA is also not *required* for centromere formation (Alonso et al. 2007; Hasson et al. 2013; Lo et al. 2001) as most neocentromeres identified in human patient samples are devoid of satellites. Further complicating a standardized model for satellites as a requisite for centromere identity, rapid evolution of centromeric satellite sequences has been observed across metazoan lineages. This rapid evolution is attributed to processes such as molecular drive, leading to the homogenization and fixation of a variant (or subset of variants) across a repeat array (Dover 1982; Dover et al. 1982), and both genetic conflict (Malik and Henikoff 2009) and centromere drive (Henikoff et al. 2001; Henikoff and Malik 2002; Malik and Henikoff 2002), leading to rapid diversification of repeat families between species. Rather than a strictly genetic model for centromere determinance, it has been proposed that eukaryotic centromere identity is maintained epigenetically through a specific histone replenishment pathway: the centromeric histone, CENP-A, loading cascade (Karpen and Allshire 1997), wherein CENP-A nucleosomes mark the centromeric region for subsequent kinetochore assembly and are replenished every cell cycle to ensure epigenetic marks for centromere function are properly inherited.

This hypothetical framework presents a conundrum—how is centromere identity maintained along evolutionary timescales and particularly during karyotypic change? Comparative studies of chromosome synteny among species, within a phylogenetic context, have revealed that centromere location on homologous chromosomes may change with no concomitant change in DNA marker order. These cases are essentially neocentromeres that have become fixed in a species, referred to as *evolutionary new centromeres*, *ENC*, most often with an accompanying expansion of satellites at the new centromere location and loss of large satellite arrays at the former location. It should be noted that while these centric shifts, or ENCs, have been identified in many different lineages, including insects, birds, and mammals (Guerra et al. 2010; Marshall et al. 2008; O'Neill et al., 2004; Schneider et al. 2016; Scott and Sullivan 2014; Tolomeo et al. 2017), some may be due to the inheritance of neocentromeres (Amor et al. 2004) while some may be the product of successive pericentric inversions (Brown and O’Neill 2010).

**Fig. 2:**
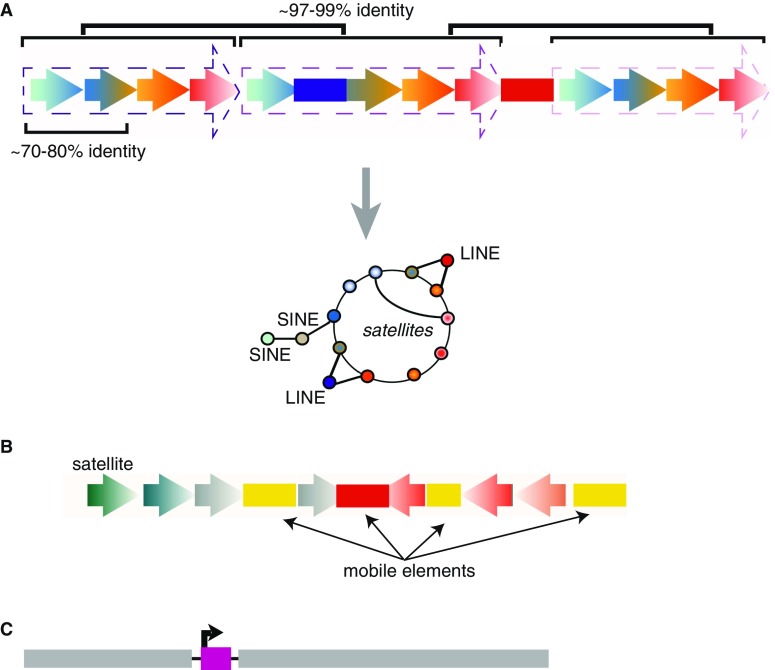
**a** (Top) The structure of a centromere following homogenization of a stable satellite (gradient arrowheads) results in arrays of satellites, each sharing 70–80% identity, which are then organized in a tandem higher order array, with each block of satellites (dotted arrowheads), known as a HOR, sharing 97–99% identity. Random insertions of TEs (colored bars) are found interspersed among the HORs. (Bottom) Illustration of the graphical map of the same centromere shown in A, with bubbles on the inner circle representing each monomer satellite and how it is arranged in relation to other monomers in the array. Gradient bubbles correspond to gradient arrowheads. Lines indicate respective satellite or TE neighbor for each satellite. TE insertions and their relative location with respect to specific monomers are indicated by solid bubbles linked to the inner circle. **b** The structure of a complex centromere, exemplified by maize, rice, and potato, is characterized by diverse TEs (colored bars) and variable satellites (gradient arrowheads). **c** The structure of a neocentromere in which a single transcriptionally active mobile element (pink) inserted into non-centromeric DNA (gray). Arrowhead indicates promoter activity

It bears noting that human neocentromeres have been shown to form at “hotpots” on certain chromosomes in the human karyotype, which often are also fragile chromosomal sites known for common occurrences of DSBs (Hasson et al. 2011). A similar “hotspot” preference for ENCs has been found in other species when synteny is considered across the phylogeny. For example, comparative sequence analysis in the tammar wallaby (*Macropus eugenii*) of a latent centromere site, an evolutionary breakpoint associated with previous centromere activity and the potential for new centromere formation (Ferreri et al. 2005; Ferreri et al. 2004), revealed an enrichment for LINEs and endogenous retroviruses at this breakpoint (Longo et al. 2009). Moreover, the orthologous human evolutionary breakpoint (14q32.33) has maintained a similar repetitive content to tammar despite last sharing a common ancestor > 150 million years ago. Evolutionary breakpoints, such as 14q32.33, are associated with chromosomal rearrangements/translocations and a subset is known to form neocentromeres (Longo et al. 2009; Ruiz-Herrera et al. 2006). It is thus possible that the presence of active TEs in such genomic regions could contribute to the instability at these evolutionary breakpoints and concomitantly to neocentromere formation. In support of this model, a human neocentromere on chromosome 10, devoid of canonical satellites, was found to carry an active transcript for a single LINE1 (Chueh et al. 2009) (Fig. 2C). This LINE1 non-coding RNA was incorporated in the neocentromeric CENP-A chromatin and was essential for the chromatin remodeling involved in the neocentromerization process. Although rare in humans, neocentromere formation does occur at a frequency of approximately one in every 70,000–200,000 live births (Marshall, et al. 2008). While their frequency in wild populations of eukaryotic species is unknown, neocentromeres can provide an effective mechanism for repositioning of the centromere and therefore can provide novel chromosome changes that can influence for karyotype evolution and chromosomal speciation (Brown and O'Neill, 2010).

The observations that satellite DNA is neither sufficient nor required, yet is virtually ubiquitous at regional centromeres across eukaryotes, even following fixation of novel centromere locations, prompt closer attention to sequences that are found in both neocentromeres and native centromeres: TEs. The emergence of massively parallel sequencing technologies and the development of over 100 different sequencing applications (“− seq”) have revealed much about the non-coding regions of the human genome interspersed across chromosome arms. While these advances have led to breakthroughs in understanding the genomic landscape for 80–90% of the human genome, the complex repeat structure of centromeres has relegated these chromosome regions to the last frontier of the human genome. Despite this, a recent and remarkable computational effort has led to the production of graphical models of human centromere sequences (Miga 2015; Miga et al. 2014; Rosenbloom et al. 2015), bypassing the need for strict linear assembly in the assessment of nascent genetic content. These “maps” (Fig. 2A) do not delineate the order of sequences within any given centromere, yet reveal the diversity of satellites within and among centromeres, supporting earlier work demonstrating that while satellite higher order repeats (HORs) are homogenized through processes such as molecular drive and concerted evolution (Dover 1982; Dover et al. 1982) (Fig. 2A), some satellites are in fact distinct among different chromosomes. Moreover, several chromosomes have multiple HORs with only one of these epialleles functioning as the active centromere (Maloney et al. 2012). As the quality of sequencing and gap-filling for the human genome increases, novel annotation workflows have uncovered retroelements scattered throughout active centromere regions across all human chromosomes, within HORs and between epialleles (Miga 2015; Rosenbloom et al. 2015).

The finding that human centromeres contain retroelements is not simply a recent discovery. Indeed, the first centromere-pericentromere boundary sequenced for human, the X chromosome, revealed that not only are retroelements present throughout, there was evidence that older elements resided farther from the core of the centromere, while recently inserted, and in some cases still active, retroelements were found within the higher order array of the centromere core (Schueler et al. 2001). Examples of the first complex eukaryotic centromeres that had been fully mapped and assembled into contiguous sequence are the small centromeres, Cen4, Cen5, and Cen8, of rice (Yan et al. 2005). Sequencing data analysis of Cen4, Cen5, and Cen8 showed that CentO satellites and centromeric retroelements (CRs) reside within the kinetochore-binding region of these centromeres (Nagaki et al. 2004; Nagaki et al. 2005). In maize and potato, years of work have shown that CRs are often a defining feature of these plant centromeres (for examples see Gent et al. 2017; Gong et al. 2012; Piras et al. 2010; Schneider et al. 2016; Zhang et al. 2014) (Fig. 2B). Fiber FISH experiments in mice showed that there are intervening sequences of unknown identity within both the maSAT and miSAT arrays (Kuznetsova et al. 2006); thus, it is likely that TEs exist within murid centromeres as they do in most complex eukaryotic centromeres. Recently, human population studies revealed that active insertions of TEs, in this case HML2, into centromeres have occurred during the evolution of modern humans and may facilitate rare centromere recombination events (Contreras-Galindo et al. 2013; Zahn et al. 2015).

Comparative studies across many species are building support for the highly concordant presence of TEs in centromeres, yet direct involvement of TEs in defining centromere identity remains elusive.

Co-option of TEs, TE insertions and the genesis of tandem duplications, and ultimately satellite DNAs are likely general aspects of centromere ontogenesis (Birchler and Presting 2012; Brown and O’Neill 2010; Chueh et al. 2009; Dawe 2003; O'Neill and Carone, 2009; O'Neill et al., 2004; 1998; Wong and Choo 2004). Recent work on the karyotypic evolution of gibbons has offered a glimpse into how rapid diversification of centromeres and chromosomes can be traced to TE activity. Although gibbons diverged from other hominids only 15–18 million years ago, the species complex is characterized by highly rearranged chromosomes (Carbone et al. 2014); among the four genera of gibbons, the number of chromosomes varies from 38 to 52. The centromeres of the Eastern hoolock gibbon were found to contain a novel TE named LAVA, LINE-Alu-VNTR-Alu-like, consisting of pieces of these repetitive elements and classified as a non-autonomous composite element that can be mobilized by LINE1 (Carbone et al. 2014; Carbone et al. 2012; Meyer et al. 2016). LAVA was subsequently found within centromeres of other gibbon species, yet shows a species-specific pattern of chromosome-delimited accumulation. The observation that entire centromere regions carried a dense accumulation of a specific TE is not unique to gibbons as a similar phenomenon had been described in the wallaby species complex with a different TE, KERV (kangaroo endogenous retrovirus) (Bulazel et al. 2007; Bulazel et al. 2006; Metcalfe et al. 2007; O'Neill et al., 1998). In both cases, epigenetic dysregulation of the TE through hypomethylation led to subsequent centromere restructuring and chromosome shuffling, likely caused by initial interspecific hybridization events (Fontdevila 2005; Metcalfe et al. 2007; Meyer et al. 2016; O'Neill and Carone, 2009; O'Neill et al., 2004; 1998).

## Centromeric TEs: co-opted and tamed or recursive invaders? A tale of two paradoxes

The activity of TEs at centromeres may in fact explain two of the paradoxes that characterize eukaryotic centromeres. *The first paradox* is the rapid diversification of satellites among species (Henikoff et al. 2001) concomitant with homogenization of arrays *across* non-homologous chromosomes within a karyotype. Mechanisms such as unequal crossing over and gene conversion are not sufficient to explain the “spread” of satellites across non-homologous chromosomes (Birchler and Presting 2012), but the genesis of satellites from TE insertions offers a possible explanation (Ahmed and Liang 2012; Mestrovic et al. 2015; Satovic et al. 2016).

A prime example of the birth of satellites from TEs can be found in *Tetris*, a novel non-autonomous foldback transposon discovered in *Drosophila virilis* and *D. americana* using in silico techniques (Dias et al. 2014). *Tetris* consists of three domains; one of which is an intermediate outer domain containing TIRs made up of ~ 220-bp internal tandem repeats (TIR-220). Interestingly, satellite DNA arrays were found that consist of TIR-220 repeats, thus demonstrating the potential ability of a TE to contribute to the formation of satellite arrays through the production of internal tandem repeats via its foldback mechanism (Dias et al. 2014).

What is less clear is whether TEs are the progenitor of *all* centromeric satellites, or if they provide another source of satellite diversification following insertion into an existing satellite-rich region (in other words, is the TE the “chicken or the egg”?). Recent work in two *Arabidopsis* species in which centromere-enriched retroelements are found indicates that specific TEs preferentially insert into centromeric regions. The ATCOPIA93 retroelement was found in low copy number scattered throughout the genome in *A. thaliana*, whereas retroelements related to ATCOPIA93 in *A. lyrata* displayed a high copy number specifically enriched in the centromeric regions (Birchler and Presting 2012; Tsukahara et al. 2012). This observation begs the question: why do homologous retroelements have distinct, and often different, genomic distributions in different genomes? Birchler and Presting (2012) suggest two possible answers to this question: (1) differing genetic and cellular environments between even closely related species influence TE integration mechanisms and/or (2) even between homologous elements, TEs rapidly diverge in their integration preference such that only one TE specifically inserts into the centromere. Tsukahara et al. (2012) performed a study where an ATCOPIA93-related element in *A. lyrata*, Tal1 (Transposon of *Arabidopsis lyrata* 1), was transformed into *A. thaliana* to test whether this TE would preferentially insert into the centromere regardless of host genome environment. Whole-genome sequencing following transformation indicated that (1) new Tal1 insertions were found in the centromeric satellite arrays of the *A. thaliana* genome and (2) the sequences flanking the inserted elements were biased towards these centromeric satellite arrays. At face value, it would reason that the Tal1 TE targets centromeric regions by recognizing satellite arrays *specifically*. However, the satellite sequences between these two species share only ~ 70% identity (Kawabe and Nasuda 2005), indicating that this is likely not a contributing factor. While it may appear that the condensed chromatin state of these centromeres, marked by DNA methylation, may provide the substrate recognized by this TE (Yamagata et al. 2007), Tal1 retained its integration preference into centromeric regions even when the overall DNA methylation in the genome was reduced via a *ddm1* mutation (Yamagata et al. 2007). So, while the epigenetic state of the centromere may play a role in site selection of TEs, it is more likely that recognition of CENP-A or other conserved centromeric proteins plays a bigger role.

**Fig. 3:**
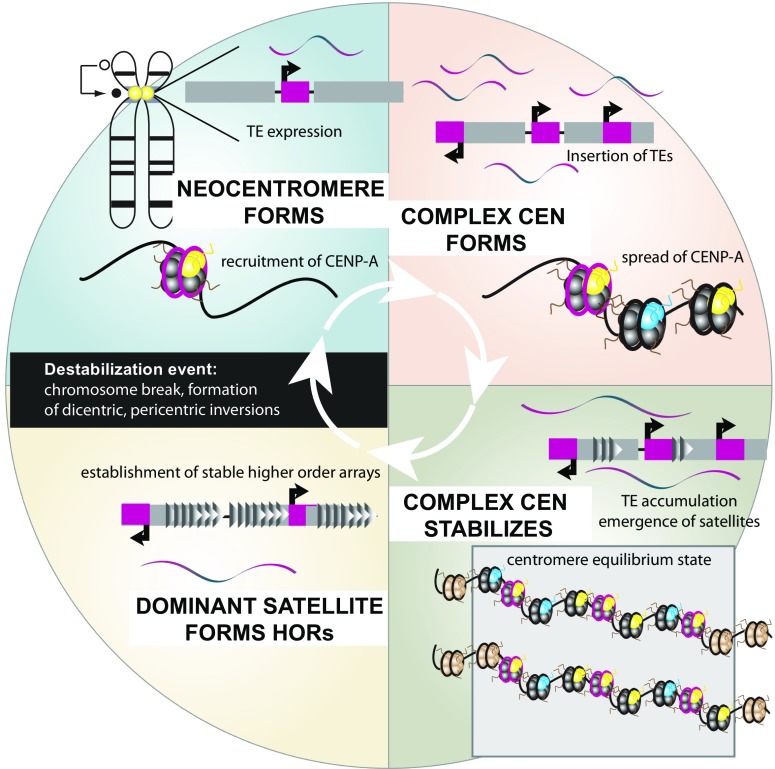
TEs and the evolution of centromeres. An initial destabilization event leads to the formation of a neocentromere (black dot indicates new centromere location, open circle indicates former centromere location on an ideogram representation of a chromosome), linked to the transcription of a TE (purple) in the absence of satellite DNA (gray). Following recruitment of CENP-A nucleosomes (yellow), more TEs insertions occur and incorporation of CENP-A nucleosomes (yellow, other H3-containing nucleosomes are indicated by blue) spread to form a complex centromere. As the complex centromere establishes an equilibrium state, TEs accumulate and satellites (arrowheads) begin to emerge. While individual variation in the placement of CENP-A nucleosomes (CENP-A containing nucleosomes are yellow, other centromeric H3-nucleosomes are blue, non-centromeric nucleosomes are brown) can exist within a population, the average centromere domain is relatively stable. At this stage of centromere evolution, interchromosomal movement of TEs can influence homogenization of arrays across non-homologous chromosomes. Finally, a dominant satellite emerges that subsequently forms higher order arrays with only intermittent TE insertions. Following a chromosome destabilization event, the HOR is either inactivated by unknown mechanisms, or lost due to chromosome damage, and a new centromere emerges in a different location

A recent study of 26 different maize lines demonstrated that following selection for centromere-linked genes and subsequent inbreeding, centromeres evolved at a rapid pace, often involving TE accumulation (Schneider et al. 2016). In some inbred lines, chromosomes were found to incorporate centromeric histones at sites *adjacent* to canonical centromere locations and in the absence of the typical tandemly arrayed satellite (CentC). Following the formation of these neocentromeres, an invasion of CR2s, a centromere-specific retroelement, followed at a frequency that established CR2-rich neocentromeres (Schneider et al. 2016) (Fig. 3). These observations further support the idea that satellites alone may not be the preferred target for CRs, rather the presence of centromeric histones and other centromeric proteins or chromatin conformation confers insertion preference for some TEs.

Another possible, but not mutually exclusive, explanation for the insertion of TEs into centromeres is that these chromosomal regions likely represent genomic “safe” insertion zones, for both the host and the TE (Birchler and Presting 2012; Sultana et al. 2017). The centromere typically encompasses a large genomic locus, is gene-poor, and consists of many repeat arrays, only some of which contain CENP-A nucleosomes; thus, it is a large genomic region into which a TE insertion would likely not cause insertional mutagenesis as surrounding repeat sequences can act as a “buffer.” Moreover, the suppression of crossing-over at the centromere would protect recently inserted retroelements from the type of recombination events that cause mutations that often result in loss of mobility. In fact, the chromosomes of some species, such as maize and potato, have an assortment of different centromeres across the karyotype, some with little satellite DNA and a variety of retroelements, many of which show variation in patterns of centromeric histone localization (Gent et al. 2017; Gong et al. 2012; Piras et al. 2010; Zhang et al. 2014), further reinforcing the observation that a single sequence does not dictate centromere identity. Rather, Gent et al. proposed that centromere positions are stably maintained, despite evidence of localized variation, as a consequence of the constraint imposed by the overall genetic landscape of the centromere (Gent et al. 2017). In a situation analogous to a “grape-in-a-bowl,” centromere position, i.e., the grape, is determined by equilibrium points on the chromosome, i.e., the bowl. In this analogy, a grape *inside* a bowl represents a “stable equilibrium position” for a centromere, affording small-scale variation in position while maintaining a stable average position across a population (Gent et al. 2017) (Fig. 3). Under such an equilibrium model, TE insertions would be buffered by an overall genetic landscape that provides a stable centromere position.

While the features that define this genetic landscape are unknown, transcription is emerging as a key component of the centromere histone replenishment pathway (Chen et al. 2015). The ability for centromeric TEs to produce non-coding RNAs provides an explanation for *the second paradox* found in centromere biology: strict inheritance of a purely epigenetic feature of the chromosome. While the finding that neocentromeres are satellite-free prompted the theory that centromeres are determined through an epigenetic process via cyclical CENP-A nucleosome deposition, neocentromeres also revealed that CENP-A deposition involved coordinated action of histone proteins and a TE non-coding RNA (Chueh et al. 2009) (Fig. 2C and Fig. 3). Similarly, our earlier work identified destabilization of centromeres in interspecific kangaroo hybrids involving activation of resident retroelements (in this case an endogenous retrovirus) (Metcalfe et al. 2007; O'Neill et al., 1998). Building on this work, we discovered a novel class of small RNAs in mammals that are derived from CRs (crasiRNAs, centromere repeat-associated short interacting RNAs) and impact the CENP-A loading cascade (Carone et al. 2008; Carone et al. 2013). We proposed that the driven elements in the centromere drive model are not simply the satellites, but the RNA-spawning elements found within centromeres: *retroelements*. These selfish entities may be the progenitors of satellite arrays that experience accretion and diminution as either monomers or large homogenous arrays following centromere stabilization and fixation in a population. In addition, retroelements provide a reasonable mechanism for the apparent concerted evolution of centromere sequences across non-homologous chromosomes. More importantly, TEs provide the means of promoting transcription within centromeres and across satellites, nascent centromeric sequences that do not otherwise carry their own promoter. For example, the CR of rice (CRR) elements are actively transcribed (Neumann et al. 2007), with centromeric satellite transcripts also identified in *Arabidopsis* (May et al. 2005), maize (Topp et al. 2004), mouse, human, and many other eukaryotic species (Ugarkovic 2005). While prevalent in complex eukaryotic centromeres, the importance of these retroelements and satellite-derived transcripts to centromere function is only recently becoming apparent: chromosome missegregation has been associated with aberrant satellite transcription in animals (Carone et al. 2008; Carone et al. 2013; Quenet and Dalal 2014; Ting et al. 2011) and satellite RNA has been implicated in the assembly of centromere components CENP-A and -C, in *Drosophila*, plants, mouse, and human (Bergmann et al. 2011; Carone et al. 2013; Chen et al. 2015; Mejia et al. 2002; Quenet and Dalal 2014; Rosic et al. 2014).

The work performed on human artificial chromosomes (HACs) has shown that while TE-free satellite arrays can support centromere function, active transcription is still a requisite for the stable propagation of the HACs (Bergmann et al. 2012; Bergmann et al. 2011; Nakano et al. 2008; Okamoto et al. 2007). HACs are typically designed to include selectable marker genes (i.e., *neo* and *bsr*) under strong, constitutive promoters juxtaposed to the α satellite arrays. Notably, centromere function of the HAC is reliant on transcriptional activity of these markers (Okamoto et al. 2007), although the need to select for cells that maintain the HAC precludes removal of the marker while maintaining efficient HAC stability. More recent work in which tetO transcriptional regulatory sequences were incorporated into HAC α satellite arrays demonstrated that a delicate balance of transcriptional activity was necessary for proper centromere function (Nakano et al. 2008). Moreover, tethering a lysine-specific demethylase (LSD1) to HAC α satellite arrays led to depletion of H3K4me2 from HAC centromeric chromatin, a loss of satellite transcription and ultimately a reduction in loading newly synthesized CENP-A (Bergmann et al. 2011). A similar targeting strategy that increased HAC centromeric H3K9 acetylation, a mark permissive to transcription, showed that a dramatic increase in transcription resulted in rapid centromere inactivation through loss of CENP-A loading on the HAC (Bergmann et al. 2012). It is possible that the ability to facilitate transcription of HAC α satellite DNA, either through a nascent promoter from a selectable marker and/or tethering factors to modulate transcription, is a proxy for what occurs natively in centromeric chromatin through the promotion of transcription via TEs.

It is clear that centromeres are rapidly evolving and thus are found in nature at many different points along their lifecycle. At each point, we observe their dynamic nature as well as their intimate relationship with TEs in their host genome. Upon initial formation as a neocentromere following some chromosomal insult or rearrangement, a single TE may serve to initiate centromere histone recruitment via its nascent transcription. As centromeric histones spread to form a centromere within an equilibrium state, a complex centromere evolves that is characterized by retroelement insertions and the evolution of divergent satellites from newly inserted centromeric TEs (Fig. 3). The genetic, epigenetic, and transcriptional landscape of these complex centromeres is a favored site of TE insertion for some elements, albeit in a species- and TE-specific, and likely target sequence agnostic, manner. An efficient and stable satellite may eventually emerge from such centromeres that enable the establishment of a highly stable centromere consisting of higher order arrays of satellites, perhaps maintained by long-range transcription from local TEs (Fig. 3). Throughout these dynamic evolutionary processes of centromere establishment, maintenance, and stabilization, TEs are a constant companion. Despite the growing evidence that this partnership pivots between the selfish propagation of TEs and the “taming” of TEs to serve a critical function of chromosome inheritance, the ongoing conflict between TEs and host genomes has found a balance that allows for the continued existence and evolution of TEs.

## Abbreviations

TE: transposable element
LTR: long terminal repeat
LINE: long interspersed nuclear element
SINE: short interspersed nuclear element
SVA: SINE-VNTR-*Alu*
VNTR: variable number tandem repeat
HERV: human endogenous retrovirus
UTR: untranslated region
ORF: open reading frame
RNP: ribonuclear protein
EN: endonuclease
RT: reverse transcriptase
TPRT: target primed reverse transcription
TSD: target site duplication
piRNA: piwi interacting RNA
CENP: centromere protein
H3: histone 3
Ddm1: decrease in DNA methylation 1
dsRNA: double-stranded RNA
RNAi: RNA interference
siRNA: short interfering RNA
RISC: RNA-induced silencing complex
FCMD: Fukuyama muscular dystrophy
NAHR: non-allelic homologous recombination
IR: inverted repeat
DSB: double strand break
TIR: terminal inverted repeat
miSAT: minor satellite
ENC: evolutionary new centromere
HOR: higher order array
CR: centromeric retroelement
CRR: centromeric retroelement of rice
LAVA: LINE-ALU-VNTR-ALU like
KERV: kangaroo endogenous retrovirus
Tal1: transposon of *Arabidopsis lyrata* 1
crasiRNAs: centromere repeat-associated short interacting RNAs
KRAB: Krüppel-associated box
KZFP: KRAB-zinc finger protein
ES: embryonic stem
TRIM28: tripartite motif containing 28
HAC: human artificial chromosome
